# Impact of Body Composition on Postoperative Outcomes in Patients Undergoing Robotic Nipple-Sparing Mastectomy with Immediate Breast Reconstruction

**DOI:** 10.3390/curroncol29010031

**Published:** 2022-01-13

**Authors:** Jiae Moon, Jeea Lee, Dong Won Lee, Hye Jung Shin, Sumin Lee, Yhenseung Kang, Na Young Kim, Hyung Seok Park

**Affiliations:** 1 Department of Anesthesiology and Pain Medicine, Anesthesia and Pain Research Institute, Yonsei University College of Medicine, Seoul 03722, Korea; answldo@yuhs.ac; 2 Department of Surgery, Yonsei University College of Medicine, Seoul 03722, Korea; jeealee84@gmail.com; 3 Department of Plastic and Reconstructive Surgery, Yonsei University College of Medicine, Seoul 03722, Korea; xyphoss@yuhs.ac; 4 Biostatistics Collaboration Unit, Department of Research Affairs, Yonsei University College of Medicine, Seoul 03722, Korea; hjshin105@yuhs.ac; 5 Department of Anesthesiology and Pain Medicine, Anesthesia and Pain Research Institute, National Health Insurance Service Ilsan Hospital, Goyang 10444, Korea; SUMINEE4946@yuhs.ac (S.L.); kmsp131@hanmail.net (Y.K.)

**Keywords:** breast cancer, robotic, nipple-sparing mastectomy, postoperative outcome

## Abstract

Nipple-areolar complex (NAC)-related complications are common during nipple-sparing mastectomy (NSM), with obesity as a risk factor. Although the incidence of NAC-related complications after robotic NSM (RNSM) with immediate breast reconstruction (IBR) is lower than that after conventional NSM, it remains one of the most unwanted complications. We aimed to evaluate body composition-based risk factors for NAC-related complications after RNSM with IBR. Data of 92 patients with breast cancer who underwent RNSM with IBR using direct-to-implant or tissue expander from November 2017 to September 2020 were analyzed retrospectively. Risk factors for NAC-related complications were identified with a focus on body composition using preoperative transverse computed tomography at the third lumbar vertebra level. Postoperative complications were assessed for 6 months. The most common complication was NAC ischemia, occurring in 15 patients (16%). Multivariate analysis revealed a low skeletal muscle index/total adipose tissue index (SMI/TATI) ratio as an independent NAC ischemia risk factor. An increase in the SMI/TATI ratio by one decreased the incidence of NAC ischemia by 0.940-fold (*p* = 0.030). A low SMI/TATI ratio is a risk factor for postoperative NAC ischemia in patients undergoing RNSM with IBR for breast cancer. Preoperative body composition-focused evaluation is more valuable than simple body mass index assessment.

## 1. Introduction

Nipple-areolar complex (NAC) ischemia and necrosis are common complications after nipple-sparing mastectomy (NSM) with a prevalence range of 0–48%, mostly 10–15% [1,2,3,4]. Several risk factors for NAC-related complications after a mastectomy with breast reconstruction (BR) have been identified, including obesity, ptosis, large breast weight and volume, prior radiotherapy history, and incision type [1,5,6,7,8], among which, obesity is a well-known risk factor [9,10,11]. Obesity is evaluated using body mass index (BMI), which reflects only body height and weight but does not reflect body composition, such as muscle and adipose tissue compositions. A risk factor analysis of current research, especially obesity, with BMI may be limited as it may not account for exact fat and muscle mass composition. Current research shows that body composition can influence mortality and survival in breast cancer patients, so it is important to assess fat and muscle mass [12]. Furthermore, a recent study reported that patients with high adipose tissue mass and low skeletal muscle mass (SMM) were more likely to develop complications after expander BR surgery [9].

Robotic NSM (RNSM) with immediate breast reconstruction (IBR) has been widely practiced worldwide since its inception in 2015 by Toesca et al. [13]. RNSM has been reported to have a lower incidence of NAC ischemia and necrosis than conventional NSM, possibly because RNSM has a vascular advantage [13,14,15,16,17,18]. However, NAC ischemia and necrosis are still predominantly undesirable complications because they significantly impact the postoperative esthetic outcome and patient satisfaction as they can cause nipple deformation, hypopigmentation, or NAC loss [1,8].

Although several studies have investigated the risk factors for NAC-related complications after NSM [1,6,7,19,20,21], no study has evaluated these risk factors after RNSM with IBR. Therefore, the purpose of this study was to investigate the risk factors for NAC-related complications after RNSM with IBR using direct-to-implant (DTI) or tissue expander, while focusing on body composition.

## 2. Materials and Methods

### 2.1. Patient Population

**Figure 1:**
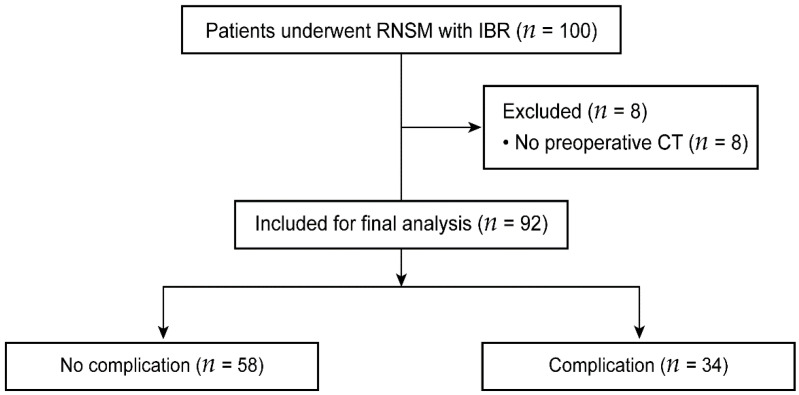
Consolidated standards of reporting trials flow diagram. RNSM, robotic nipple-sparing mastectomy; IBR, immediate breast reconstruction; and CT, computed tomography.

This study was approved by the institutional review board (IRB) and hospital research ethics committee of Yonsei University Health System, Seoul, Korea (IRB protocol No. 4-2021-0585). Patient information was anonymized prior to analysis, and the prerequisite for obtaining informed consent was waived. We retrospectively reviewed the electronic medical records of 100 consecutive patients with breast cancer who underwent RNSM with IBR using DTI or tissue expander from November 2017 to September 2020. In our institution, RNSM with implant-based IBR was performed on selected patients with small- to medium-sized breasts and without ptosis. Eight patients who did not undergo preoperative abdominal computed tomography (CT) or whole-body positron emission tomography-CT were excluded from the analysis. Finally, 92 patients were included and analyzed in the current study (Figure 1).

### 2.2. Procedures

Details of the surgical techniques for RNSM have been described elsewhere [13,14,16,22,23]. RNSM was performed through a 2.5–6 cm mid-axillary incision. The working space under the skin flap or retromammary space was developed manually. After docking the robotic surgical system, dissection and the entire breast parenchyma retrieval were performed through the same incision. Immediate reconstruction was achieved using DTI or tissue expander in the sub- or pre-pectoral space.

### 2.3. Complications

The incidence of postoperative complications was assessed for 6 months after surgery. In this study, NAC ischemia was graded according to the surface area of the ischemic breast tissue using the classification method proposed by Ahn et al. [1]: no ischemia (grade 0), partial nipple or areolar ischemia (grade 1), partial NAC ischemia (grade 2), total nipple ischemia (grade 3), total nipple and partial areolar ischemia (grade 4), and total NAC ischemia (grade 5). NAC ischemia was defined when any part of the NAC exhibited clinical ischemic color changes that were resolved by conservative management, such as the application of moist dressing or topical ointment. NAC necrosis was defined as the occurrence of full-thickness necrosis of the NAC requiring surgical treatment. NAC loss was defined as nipple loss following NAC ischemia and necrosis. In addition, the occurrences of postoperative complications, including skin ischemia or necrosis, implant loss, wound dehiscence, seroma, and infection were assessed.

### 2.4. Body Composition Assessment on CT Images

The SMM, as well as subcutaneous, visceral, and total adipose tissue areas, were measured by a radiologist at the 3rd lumbar vertebra (L3) level of a transverse, cross-sectional CT image obtained during preoperative staging workup [24]. Total cross-sectional areas were measured by applying Hounsfield unit thresholds of −29 to +150 for skeletal muscle, −190 to −30 for subcutaneous adipose tissue, and −50 to −150 for visceral adipose tissue, using a commercially available imaging software (Aquarius Intuition version 4.4.12, TeraRecon Inc., San Mateo, CA, USA) [25,26]. The measured areas (cm^2^) were normalized for height (m^2^) and were defined as the skeletal muscle index (SMI), subcutaneous adipose tissue index (SATI), visceral adipose tissue index (VATI), and total adipose tissue index (TATI). The SMI/TATI ratio was calculated for each patient.

### 2.5. Data Collection

We collected the following patient demographic and clinical data: age, smoking history, American Society of Anesthesiologists physical status, underlying diseases (such as hypertension or diabetes mellitus), menopause status, neoadjuvant chemotherapy, BMI, SMI, SATI, VATI, and TATI. The following intraoperative variables were analyzed: surgical extent, axillary lymph node dissection, type of reconstruction, other combined surgical procedures, specimen weight, duration of anesthesia and operation, intraoperative blood loss, dose of remifentanil administered, intraoperative urine output, and postoperative hospital stay. Moreover, postoperative complications that occurred within 6 months were investigated.

### 2.6. Statistical Analysis

For continuous variables, the normality test (Shapiro–Wilk test) was performed. If normality was satisfied, descriptive statistics were expressed as mean ± standard deviation, and the Student’s *t*-test was used to compare the difference between the two groups. The variables that passed the normality test were age and specimen weight. For continuous variables that did not pass the normality test, descriptive statistics were expressed as median (first to third quartile (Q1–Q3)), and the Mann–Whitney U test was used to compare group differences. For categorical variables, descriptive statistics were expressed by the number of patients (percentage), and the chi-squared or Fisher’s exact test was used to compare the two groups.

Odds ratios (ORs) and 95% confidence intervals (CIs) of the potential risk factors for NAC-related complications were obtained through univariate logistic regression analyses. Variables that showed significant differences (*p* < 0.05) in the univariate analysis were considered independent variables in the multivariate analysis to evaluate the risk factors for NAC ischemia. A forest plot of ORs and 95% CIs was produced to visually represent the association between risk factors and NAC ischemia.

## 3. Results

Table 1 demonstrates the demographic and preoperative characteristics of the enrolled patients. The average BMI and SMI of total patients were 22.0 (range, 20.2–23.0) kg/m^2^ and 39.7 (range, 36.4–43.4) cm^2^/m^2^, respectively. No differences were found in these variables between the no-complication and complication groups. The SATI, VATI, and TATI were significantly higher (*p* = 0.007, <0.001, and <0.001, respectively), whereas the SMI/TATI ratio was significantly lower in the complication group (*p* < 0.001) than in the no-complication group.

The operative variables are presented in Table 2. The median specimen weight was 350 (range, 263–440) g, which was significantly higher in the complication group than in the no-complication group (*p* = 0.004). The complication group tended to have a longer duration of mastectomy; however, there was no statistical significance between-group difference. Furthermore, there was no between-group difference in the other variables.

Table 3 presents the NAC ischemic grade and the overall complication rate. Of the 92 patients included in the study, 34 (37%) had one or more complications. The most common complication was NAC ischemia requiring conservative treatment, which occurred in 15 patients (16%), of whom seven had ischemia grade 1, five had grade 2, and three had grade 3. Four patients (4%) with NAC necrosis underwent surgical intervention, of whom one had ischemic grade 3, two had grade 4, and one had grade 5. NAC loss occurred in two patients (2%).

Univariate analysis was performed for NAC ischemia, and the OR and 95% CIs obtained for each variable are shown in Table 4. The following variables were potential risk factors for NAC ischemia: high BMI, high SATI, high VATI, high TATI, high specimen weight, and low SMI/TATI ratio.

**Figure 2:**
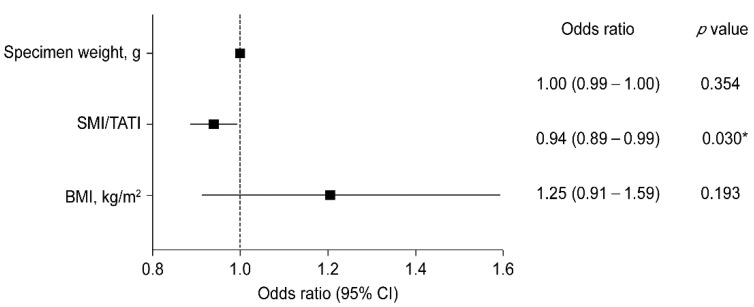
Forest plot of multivariate logistic regression model analysis for NAC ischemia. CI, confidence interval; SMI, skeletal muscle index; TATI, total adipose tissue index; and BMI, body mass index. * *p* < 0.05.

Multivariate logistic regression revealed that a low SMI/TATI ratio was a significant risk factor for NAC ischemia (Figure 2). A unit increase in SMI/TATI ratio resulted in a 6% reduction in the odds of NAC ischemia occurrence when the specimen weight and BMI were controlled (*p* = 0.030).

## 4. Discussion

In this retrospective study, we evaluated the risk factors for NAC-related complications after RNSM with IBR using DTI or tissue expander, with a focus on body composition. NAC ischemia occurred in 16% of patients in the current study, and a low SMI/TATI ratio significantly increased the incidence of NAC ischemia after RNSM with IBR.

NAC ischemia and necrosis reportedly occur in 0–13% and 0–2.4%, respectively, of patients after RNSM with IBR [15,16,18,22,27,28]. NAC ischemia and necrosis can be caused by alterations in breast vascularity. Although the blood supply of the breast is unpredictable and variable, both the lateral thoracic and internal mammary arteries are important vessels supplying the NAC. If the blood supply or venous drainage network of the NAC is interrupted, vascular-dependent complications such as ischemia and necrosis may occur [29,30]. During RNSM, the incision is made away from the breast in the axillary line, which may have contributed to reducing the blood supply interruption [14,15,16,17]. In addition, the intercostal perforators and lymphatics can be clearly recognized by the robotic optic system, thereby reducing damage to the entire circulation of the NAC [13,17]. In the present study, 15 out of 92 patients (16%) developed NAC ischemia, which was higher than that reported in previous studies, probably because patients with low-grade transient partial ischemia were also included in our study.

Several studies have demonstrated that a high BMI could be associated with NAC-related complications [7,31,32]. Moreover, patients with high BMI may be predisposed to developing microvascular dysfunction and compromised skin flap perfusion, which could increase the incidence of NAC-related complications [33,34]. In addition, patients with obesity tend to develop NAC-related complications because the surface of the mastectomy flap and the length of the skin flap between the NAC and the thoracic wall are larger than those in patients without obesity, which may affect the blood supply of the skin flap [1,6,10]. Nguyen et al. found that BMI is a continuous predictor of complications after expander-implant BR [10]. Chang et al. reported that BMI ≥ 24 kg/m^2^ is a risk factor for complications after tissue expander-based BR in patients of Asian ethnicity [33]. However, in the present study, BMI was associated with NAC ischemia in the univariate analysis but was not a risk factor in the multivariate analysis. BMI is a measure of body fat based on height and weight, but it has a limitation in that it cannot represent body composition. Nakamura et al. reported that a high fat mass and low SMM were risk factors for all complications and delayed wound healing after tissue expander BR [9]. Consistent with their findings, we found that a low SMI/TATI ratio was a risk factor for NAC ischemia, but skeletal muscle or adipose tissue, by itself, was not associated with NAC ischemia.

Breast size or specimen weight may also be associated with NAC-related complications [6,35,36,37]. Chirappapha et al. highlighted that the volume of the breast removed was a risk factor for NAC necrosis [6]. Moreover, Woo et al. reported that a larger breast size was significantly associated with the incidence of overall and major complications after immediate expander-implant BR [35]. In conventional NSM, large breasts are thought to have vascular disadvantages in terms of the skin flap and NAC areas [6]. The present study found a significant association between specimen weight and NAC-related complications in the univariate analysis but not in the multivariate analysis. This may be because the RNSM in this current study was performed on selected patients with small- to medium-sized breasts without ptosis [28], which is similar to patient recruitment in previous studies on RNSM [15,17].

In summary, in our study, BMI, adipose tissue mass, SMM, and specimen weight were not found to be associated with NAC ischemia; however, the incidence of NAC ischemia was significantly increased when the SMI/TATI ratio was low. Body composition is an important factor that affects the outcomes of patients with breast cancer; a higher SMM reportedly has a more favorable effect on mortality and overall survival in patients with early-stage breast cancer [24,38]. Furthermore, we found that the ratio of SMM relative to total adipose tissue mass affects the development of postoperative NAC ischemia. Patients with low preoperative SMI/TATI ratios should be informed about the risk of postoperative NAC ischemia. Preoperative interventions to increase the SMI/TATI ratio should be considered, and the patients should be carefully managed postoperatively. Preoperative interventions, such as muscle training and nutritional support, are thought to reduce the incidence of NAC ischemia, thereby improving the esthetic outcomes and postoperative prognosis, including overall survival and mortality [9,12,24].

This study has some limitations as its retrospective nature renders it susceptible to selection bias. In our institution, RNSM with IBR was performed on highly selected patients with early-stage breast cancer having small- to medium-sized breasts without ptosis. Moreover, this was a single-center study with a small sample size. Future large-scale, prospective randomized controlled trials are warranted to clarify the relationship between body composition and postoperative complications, to predict risk factors for postoperative NAC-related complications after RNSM, and to suggest ways to improve patient outcomes through preoperative intervention and postoperative management in patients with low SMI/TATI ratios. Despite these limitations, this is, to the best of our knowledge, the first study to evaluate the risk factors for NAC-related complications in patients undergoing RNSM with IBR. In addition, the strength of this study lies in the fact that multiple risk factors, including tobacco use, BMI, diabetes mellitus, and specimen weight, are considered. Moreover, a novel positive association between body composition and NAC ischemia is presented in addition to previously known risk factors.

## 5. Conclusions

A low preoperative SMI/TATI ratio is a significant risk factor for postoperative NAC ischemia in patients who underwent RNSM with IBR. Thus, preoperative evaluation focusing on body composition is more important than a simple BMI assessment; this furnishes more accurate information that can lead to improved postoperative patient outcomes.

## Figures and Tables

**Table 1:** Patient demographic and preoperative characteristics.

Variable	No Complication (*n* = 58)	Complication (*n* = 34)	*p*-Value
Age, years	44 ± 7	45 ± 8	0.296
Smoking history			0.605
Non-smoker	57 (98%)	33 (97%)	
Ex-smoker	0 (0%)	1 (3%)	
Current smoker	1 (2%)	0 (0%)	
ASA physical status			0.358
I	34 (59%)	16 (47%)	
II	21 (36%)	14 (41%)	
III	3 (5%)	4 (12%)	
Comorbidities			
Hypertension	3 (5%)	2 (6%)	>0.999
Diabetes mellitus	1 (2%)	1 (3%)	>0.999
Menopause status			0.690
Premenopausal	48 (83%)	27 (79%)	
Postmenopausal	10 (17%)	7 (21%)	
Neoadjuvant chemotherapy	5 (9%)	4 (12%)	0.721
Body mass index, kg/m^2^	21.3 (20.1, 22.6)	22.5 (20.4, 24.0)	0.088
Skeletal muscle index, cm^2^/m^2^	38.7 (36.4. 43.4)	40.3 (37.6, 45.0)	0.353
Subcutaneous adipose tissue index, cm^2^/m^2^	48.3 (34.9, 60.2)	58.7 (47.0, 76.4)	0.007 *
Visceral adipose tissue index, cm^2^/m^2^	15.7 (9.43, 21.1)	27.0 (17.2, 37.7)	<0.001 *
Total adipose tissue index, cm^2^/m^2^	62.9 (48.3, 85.2)	89.2 (70.1, 110.4)	<0.001 *
SMI/TATI	66.6 (47.3, 83.6)	46.3 (40.3, 55.5)	<0.001 *

Data are presented as mean ± standard deviation, number of patients (proportion), or median (first to third quartile (Q1, Q3)). * *p* < 0.05; ASA, American Society of Anesthesiologists; SMI, skeletal muscle index; and TATI, total adipose tissue index.

**Table 2:** Operative characteristics.

Variable	No Complication (*n* = 58)	Complication (*n* = 34)	*p*-Value
Surgical extent			0.367
Unilateral	47 (81%)	30 (88%)	
Bilateral	11 (19%)	4 (12%)	
ALND	11 (19%)	4 (12%)	0.367
Type of reconstruction			0.358
Direct-to-implant	41 (71%)	27 (79%)	
Tissue expander insertion	17 (29%)	7 (21%)	
Reconstruction location			0.367
Pre-pectoral	47 (81%)	30 (88%)	
Sub-pectoral	11 (19%)	4 (12%)	
Combined other operation	10 (17%)	7 (21%)	0.690
Specimen weight, g	313 (248, 395)	387 (322, 471)	0.004 *
Duration of anesthesia time, min	346.5 (300, 425)	362.5 (305, 445)	0.247
Duration of operation time, min	299 (246, 372)	305 (260, 387)	0.509
Duration of mastectomy time, min	170 (142, 214)	198 (168, 233)	0.058
Duration of reconstruction time, min	110.5 (89, 133)	122 (107, 157)	0.088
Intraoperative blood loss, mL	50 (30, 70)	50 (30, 150)	0.144
Intraoperative fluid input rate, mL/min	6.1 ± 1.5	6.6 ± 1.5	0.209
Intraoperative urine out, mL	467.5 (340, 780)	427.5 (280, 620)	0.496
Administered dose of remifentanil, mg	0.9 (0.8, 1.1)	1.0 (0.9, 1.3)	0.091
Postoperative hospital days	9 (7, 11)	10 (8, 12)	0.085

Data are presented as mean ± standard deviation, number of patients (proportion), or median (first to third quartile (Q1, Q3)). * *p* < 0.05 ALND, axillary lymph node dissection.

**Table 3:** Incidence of postoperative complications.

Variable	Number (%)
NAC	
NAC ischemic grade	
Grade 0	73 (79%)
Grade 1	7 (8%)
Grade 2	5 (5%)
Grade 3	4 (4%)
Grade 4	2 (2%)
Grade 5	1 (1%)
NAC ischemia (Resolved with conservative treatment)	15 (16%)
NAC necrosis (Required Surgical treatment)	4 (4%)
NAC loss	2 (2%)
OTHERS	
Skin ischemia or necrosis	7 (8%)
Implant loss	2 (2%)
Wound dehiscence	1 (1%)
Seroma	6 (7%)
Infection	7 (8%)

NAC, nipple-areolar complex.

**Table 4:** Univariate analyses of risk factors for NAC ischemia after RNSM with IBR.

Variable	Odds Ratio	95% CI	*p*-Value
Age, years	1.06	[0.99–1.14]	0.087
Smoking	1.07	[0.11–10.34]	0.951
Diabetes mellitus	5.43	[0.32–91.99]	0.241
BMI, kg/m^2^	1.30	[1.08–1.58]	0.007 *
SMI, cm^2^/m^2^	1.01	[0.96–1.06]	0.773
SATI, cm^2^/m^2^	1.03	[1.01–1.06]	0.018 *
VATI, cm^2^/m^2^	1.07	[1.03–1.11]	0.001 *
TATI, cm^2^/m^2^	1.03	[1.01–1.05]	0.002 *
SMI/TATI	0.93	[0.89–0.98]	0.004 *
ALND	0.32	[0.04–2.65]	0.292
Type of reconstruction			
DTI	ref		
TE insertion	0.39	[0.08–1.85]	0.233
Location			
Pre-pectoral	ref		
Sub-pectoral	0.76	[0.15–3.77]	0.734
Combined with other surgery	1.13	[0.28–4.52]	0.868
Specimen weight, g	1.00	[1.00–1.01]	0.026 *
Anesthesia duration, min	1.00	[1.00–1.01]	0.911
Operation duration, min	1.00	[0.99–1.01]	0.958
Blood loss >100mL	1.77	[0.53–5.87]	0.354
Fluid input rate, mL/kg/min	1.04	[0.72–1.51]	0.832
Urine out, mL	1.00	[1.00–1.00]	0.460
Postoperative hospital stays, days	0.96	[0.79–1.17]	0.668

NAC, nipple-areolar complex; RNSM, robotic nipple-sparing mastectomy; IBR, immediate breast reconstruction; CI, confidence interval; BMI, body mass index; SMI, skeletal muscle index; SATI, subcutaneous adipose tissue index; VATI, visceral adipose tissue index; TATI, total adipose tissue index; ALND, axillary lymph node dissection; DTI, direct-to-implant; and TE: tissue expander. * *p* < 0.05.

## Data Availability

The data presented in this study are available on request from the corresponding author.
